# Use of Nanoparticles in Regenerative Dentistry: A Systematic Review

**DOI:** 10.3390/biomimetics9040243

**Published:** 2024-04-18

**Authors:** María Pilar Pecci-Lloret, Silvia Gea-Alcocer, Laura Murcia-Flores, Francisco Javier Rodríguez-Lozano, Ricardo Elías Oñate-Sánchez

**Affiliations:** 1 Special Care in Dentistry and Gerodontology Unit, Meseguer Hospital, Faculty of Medicine, University of Murcia, 30100 Murcia, Spain; mariapilar.pecci@um.es (M.P.P.-L.); silvia.gea@um.es (S.G.-A.); reosan@um.es (R.E.O.-S.); 2 Department of Health Sciences, Catholic Unisersity San Antonio of Murcia, 30107 Murcia, Spain; lmurcia@ucam.edu

**Keywords:** nanoparticles, regenerative, dentistry

## Abstract

Introduction: nanoparticles are tiny-sized materials whose characteristics and properties mean that their association with dental materials is being investigated to ascertain their effects and possible benefits on tooth structures. This systematic review aimed to qualitatively collect in vitro studies that address the potential application of different nanoparticles in dental regeneration. Following an exhaustive search and article selection process, 16 in vitro studies that met our eligibility criteria were included. BG-NPs were analyzed across five studies, with three demonstrating their impact on the growth and differentiation of human hDPSCs. CS-NPs were examined in three studies, with findings from two indicating a significant effect on the differentiation of SCAPs. Nanoparticles’ therapeutic potential and their stimulatory effect on promoting the regeneration of cells of the dentin-pulp complex have been proven. Their effect is altered according to the type of nanoparticle, concentration, and substances associated with them and, depending on these variables, they will affect the pulp, dentine, and dental cementum differently.

## 1. Introduction

Nanoparticles, (also referred to as nanomaterials), characterized by their diminutive size ranging from 1 to 100 nanometers, exhibit unique physical, chemical, mechanical, and biological properties. These distinctive characteristics have catalyzed their widespread investigation in a plethora of research studies in recent years [1]. Nanoparticles are systematically categorized based on their origin as either natural or synthetic, with the natural category further subdivided into organic and inorganic classes. Other classifications of these can be based on their shape (cylindrical or tubular, plates or rods) or according to their dimensions (zero, one, two, or three dimensions) [2].

In the realm of dentistry, the integration of nanoparticles into dental materials has yielded advantageous outcomes across a variety of applications including periodontics, restorative and preventive dentistry, and endodontics, as well as dental and bone regeneration [3]. Specifically, in the treatment of periodontal diseases, an array of nanoparticles such as poly(dopamine); poly(D,L-lactide acid) (PLA); poly(glycolic acid); poly(D,L-lactide-co-glycolide acid) (PLGA); triclosan-loaded cellulose phthalate (TCS); and chitosan have been utilized. Notably, hydroxyapatite nanoparticles in conjunction with tetracyclines have demonstrated promising results in enhancing the health of the periodontal ligament [3,4].

In preventive dentistry, various nanoparticles including hydroxyapatite, PAMAM dendrimers, calcium phosphate, and chitosan have been identified as effective against periodontitis. Gold nanoparticles, in particular, have been highlighted for their antibacterial and antifungal capabilities, offering a treatment strategy for periodontitis and aiding in the remineralization process through varnish applications. Their role extends to bone regeneration, stimulating osteogenesis by promoting bone cell differentiation and proliferation [1,3,5].

Within endodontics, chitosan’s antibacterial properties, especially against Enterococcus faecalis, along with its biocompatibility, enable its use either alone or combined with chlorhexidine as a sealing cement. Furthermore, chitosan nanoparticles have demonstrated regenerative capabilities. Similarly, bioactive glass nanoparticles have been shown to facilitate dentine remineralization and tissue regeneration, promoting odontoblast differentiation from dental pulp stem cells and enhancing cementoblast activity for new tissue formation in the root cementum and periodontium [2,6].

Nanohydroxyapatite, closely resembling the inorganic component of bone, has been extensively employed in bone regeneration due to its potent influence on bone formation processes, applicable in surgeries or implants and beneficial for periodontium or root applications [1,2].

In the context of implants and alveolar bone applications, the employment of nanoparticles on implant surfaces has been explored to enhance osseointegration. A variety of nanoparticles including hydroxyapatite, phosphate and calcium nanoparticles, gold nanoparticles, chitosan, titanium oxide, and graphene oxide have been utilized to promote osseointegration and improve implant surface adhesion [3,7].

Dental regeneration research has underscored the necessity of growth factors, precursor cells, and specific scaffolds such as nanomaterials and nanoparticles [1,8]. These components facilitate cell differentiation and proliferation, and in some instances, angiogenesis or cell migration, aiming to replicate or fully recover the original dental tissue functionality, especially within the dentin-pulp complex. This includes achieving vascularization, innervation, apical closures, and the comprehensive integration of soft tissues [8].

Current investigations into the regeneration of the dentin-pulp complex are exploring three primary methodologies: cell transplantation with suitable scaffolds, cell-based therapies predominantly utilizing ceramic materials like MTA, and growth factor-guided regeneration therapies [8].

The main objective of this systematic review is to qualitatively gather and analyze studies that explore in vitro the application of various nanoparticles as potential agents in dental regeneration.

## 2. Materials and Methods

### 2.1. Declaration and Protocol

The following systematic review was conducted with the help of the “Preferred Reporting Items for Systematic Reviews and Meta-Analyses”, better known as the PRISMA 2020 guide [9]. In addition, it is registered in Open Science Framework (OSF) registries (osf.io/fr53z). https://doi.org/10.17605/OSF.IO/FR53Z (accessed on 16 April 2024).

### 2.2. Inclusion and Exclusion Criteria

The inclusion criteria were the following: (I) in vitro articles; (II) articles that examined and referred to how nanoparticles promote the regeneration of dental structures; (III) studies that evaluated the dental regeneration potential of nanoparticles; (IV) studies that had been performed on extracted human teeth or dental cells; and (V) studies published in English.

On the other hand, the exclusion criteria were as follows: (I) studies that merely sought to report on nanoparticles’ antibacterial and disinfection effects; (II) animal tests; (III) any study that was not in vitro; (IV) systematic reviews; and (V) a language other than English.

Eligibility criteria were established following the PIOS model:

- Patient or population (P): extracted human teeth and dental cells involved in dental regenerative procedures.
- Intervention (I): application of different nanoparticles.
- Outcomes: evidence of therapeutic potential in dental regeneration (osteoblastic differentiation, osteogenic differentiation, increase of alkaline phosphatase activity).
- Study design (S): in vitro (studies conducted with stem cells).

So, the PICO question was “whether nanoparticles successfully regenerate dental tissue?”.

### 2.3. Search Strategy

#### 2.3.1. Databases/Sources of Information

A thorough and detailed search was performed in the following databases to obtain the articles intended to be the subject of this systematic review: PubMed, Scopus, and Web of Science. The search was done on 22 November 2022, and updated on 22 March 2023 and 3 April 2024.

#### 2.3.2. Search Terms

The search terms used to search for articles containing detailed information about the topic in question for this systematic review were obtained from the Mesh thesaurus. The following terms used were used*:* “silver nanoparticles”, “nanoparticles”, “endodontic”, “root canal treatment”, and “regenerative dentistry”. Boolean operators (“AND” and “OR”) were used to unify the search for the above terms with each other. The table below shows the search results in the databases mentioned above in more detail (Table 1).

#### 2.3.3. Studies Selection

The articles obtained from the search were added to the EndNote Online bibliographic manager (Clarivate) to continue with the selection process, and duplicates were subsequently discarded.

Then, the first selection of articles was made solely based on their title and abstract, paying particular attention to compliance with the established inclusion and exclusion criteria. Finally, the studies were read in-depth and analyzed in full text to finalize their selection.

#### 2.3.4. Data Extraction

Concerning the data extraction from the articles, categories were considered for each relevant study: authors, year of publication, control group, type of study, tissue on which they act, type of nanoparticle, effect achieved, and type of application.

#### 2.3.5. Quality Evaluation/Analysis

The quality analysis of the studies included in this review has been analyzed following the guidelines of the modified CON-SORT (Consolidated Standards Of Reporting Trials) [10] checklist (Table 2), specifically for in vitro studies of dental materials, which shows a series of guidelines on what should be included in a pre-clinical trial, and this one in particular, in the field of dentistry.

## 3. Results

### 3.1. Study Selection and Flowchart

**Figure 1:**
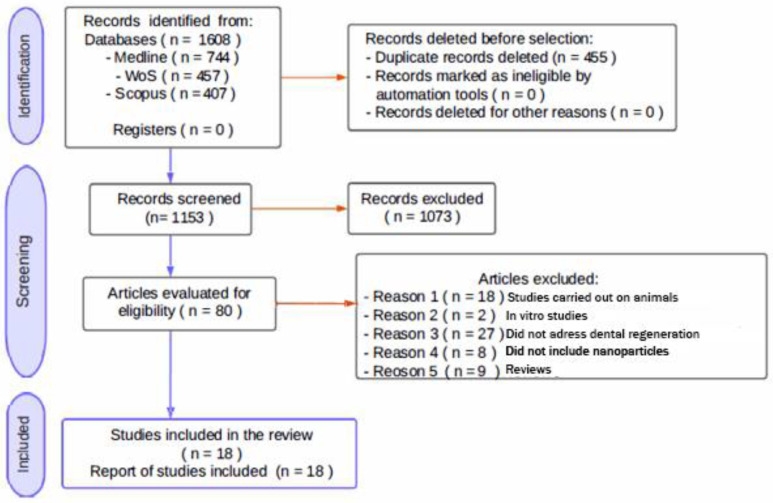
The flow chart shows the selection of studies according to Prisma 2020.

The methodology for selecting studies and the resultant data are depicted in Figure 1. An extensive search across various databases generated 1608 references, comprising 744 from Medline Pubmed, 457 from Web of Science, and 407 from Scopus. Subsequently, utilizing the Endnote^®^ Online bibliographic management tool, 455 duplicates and undetected duplicates were eliminated. This process narrowed the pool to 1153 references, which were then screened by title and abstract. As a result, 1073 references were excluded, permitting a detailed examination of the full texts of the remaining 80 studies. Within this subset, 18 studies were excluded due to their focus on animal research, encompassing both in vitro and in vivo methodologies, 2 were excluded for being in vivo human studies, 27 were disregarded for not addressing dental regeneration, 8 were eliminated for their lack of nanoparticle usage, and 9 were eliminated because they were review articles or systematic reviews. Consequently, 18 studies were deemed pertinent for inclusion in this review.

### 3.2. Data Extraction Results

The results of the data extraction are shown in Table 3. It presents the abovementioned characteristics for evaluation in the different studies and, consequently, for comparison.

### 3.3. Quality Evaluation Results

**Figure 2:**
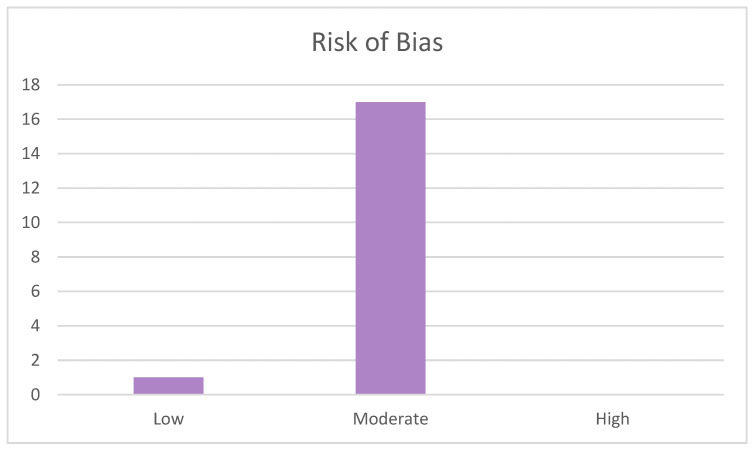
Representation of studies according to risk of bias.

Regarding the assessment of study quality, the results are reflected in Table 4 and were analyzed with the modified CONSORT checklist [10]. The quality of the studies was medium-low (Figure 2) because the majority of studies did not meet the randomization criteria referred to in items 6, 7, 8, and 9, which required the method; randomization mechanism; which investigator or participant had generated it; and who and how they had been blinded once the randomization sequence had been performed, respectively. Only in one study [15] was item 8* considered valid, as it reflected which authors had carried out each part of the study, some having developed the method and others having analyzed the results. Similarly, item 4 was not met by a number of studies [11,12,13,14,15,16,17,18,19,20,21,22,23,24,25,26] because it could only be awarded if it expressly stated “measure of outcomes”.

On the other hand, items 3, 11, and 13, referring to the structuring of the article, results and estimation, and funding, respectively, were respected by a number of articles [11,12,13,14,15,16,17,18,19,20,21,22,23,24,25,26]. Item 1, based on the structuring of the initial abstract, was not fulfilled by many articles [11,12,14,15,16,20,22,25,26]. The requirements established for the introduction were not followed by two articles [11,16] for item 2a referring to the context and justification, nor by a number of articles [12,15,20,25] for item 2b based on the objectives.

Thus, the assessment of sample size in item 5 was not met by one article [22]; the statistical method referred to in item 10 was not met by one article [12]; item 12 representing the limitations of the study was negatively assessed in many articles [11,12,13,14,16,18,20,21,22,24,25]; and finally, item 14 based on access to the trial protocol was only met by three articles [14,18,19]. Figure 2 depicts, for the purpose of synthesis, the risk of bias of the studies.

### 3.4. Bibliometric Analysis

**Figure 3:**
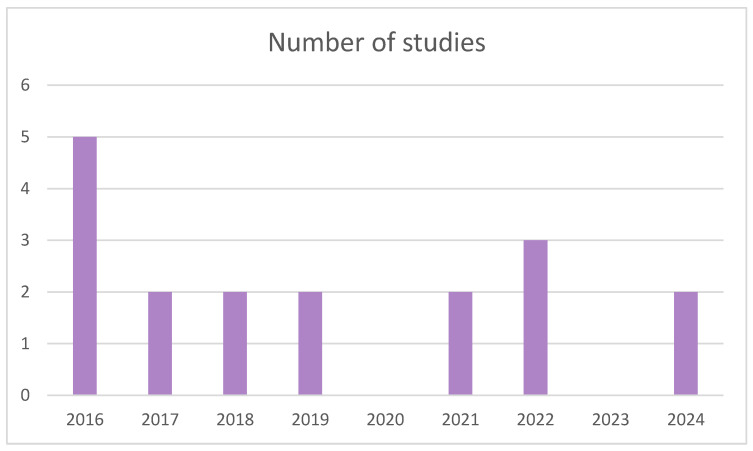
Analysis of the literature according to its year of publication.

The articles included have been classified by year, country, and journal of publication. With regard to the year of publication (Figure 3), the greatest interest in the main topic of this review is seen in 2016, with the highest frequency of publication, while the following years were less influential, with three studies published per year. The second-most prevalent year is 2022, with three publications; however, no studies were published in 2020 or 2023. Given that the effect of nanoparticles is a technology-related topic and is still under investigation today, the frequency of studies is expected to increase to obtain more information.

**Figure 4:**
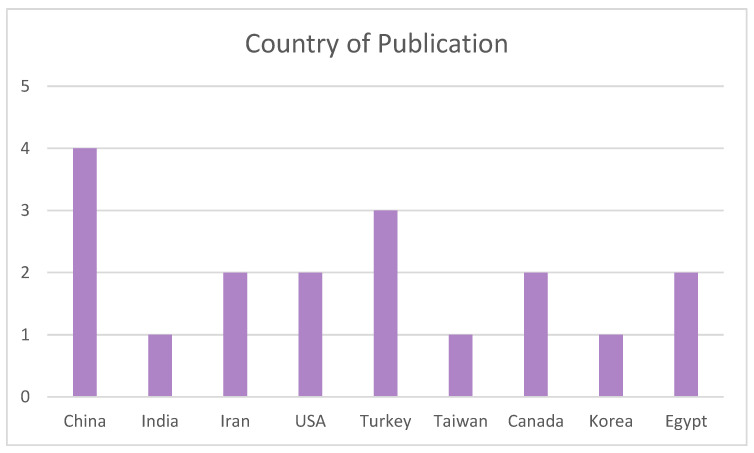
Analysis of the literature according to its country of publication.

Regarding the country of publication represented in Figure 4, it can be seen that the articles have been published in America, Africa, or Asia, especially China, which is the most prevalent with four publications, followed by Turkey with three studies. The other countries have one or two publications each.

**Figure 5:**
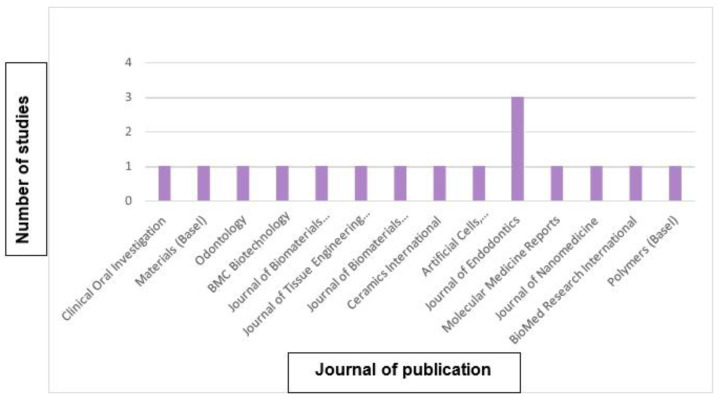
Analysis of the bibliography according to the journal of publication.

Different journals have included studies about dental regeneration, with the Journal of Endodontics publishing three articles, in contrast to the rest, which have one publication each, as shown in Figure 5.

## 4. Discussion

Initially, our examination focused on the utilization of bioactive glass nanoparticles (BG-NPs), which, as evidenced by the majority of studies included in this review, are frequently combined with boron. Moonesi Rad et al. [11] report that the incorporation of boron into bioactive glass nanoparticles results in the formation of a bilayer membrane characterized by asymmetric distribution of these nanoparticles. This modification led to favorable outcomes in terms of the differentiation of human dental pulp stem cells (hDPSCs), thereby inducing osteogenic activity and endorsing the role of these nanoparticles as a bioactive material conducive to regenerative osteogenic growth (ROG) [11]. In a parallel investigation conducted by Rao et al. [15], bioactive glass nanoparticles were amalgamated with tideglusib and integrated with calcium silicate cement, with the objective of assessing their efficacy in comparison to a control group utilizing Biodentine. The findings revealed that the combination of nanoparticle-enhanced bioactive glass with tideglusib significantly enhanced lesion healing and facilitated the proliferation of hDPSCs, thus indicating a superior therapeutic outcome.

In a subsequent study, Moonesi Rad et al. [16] explored the potential of bioactive glass nanoparticles (BG-NPs) that were modified with boron and polymers for the purpose of dentin regeneration. This innovation in NP-BG composition facilitated the formation of phosphate and calcium deposits, forming a layered structure conducive to the migration and differentiation of human dental pulp stem cells (hDPSCs), thereby demonstrating promising regenerative effects.

Similarly, Lim et al. [22] conducted a study to evaluate the effectiveness of bioactive glass nanoparticles (NP-BG) when combined with dexamethasone (DEX), aimed at promoting the differentiation of human dental pulp cells (hDPCs) via the activation of the enzyme alkaline phosphatase (ALP). The investigation revealed that the NP-BG formulation augmented with DEX exhibited superior odontogenic outcomes compared to the control group without NP-BG. Over time, the ALP levels in the NP-BG-DEX group showed a significant increase, indicating a marked overexpression of ALP when compared to the NP-BG group that did not contain DEX, thus underscoring the enhanced therapeutic potential of this composite in dental tissue engineering.

In their research, Rad et al. [25] dedicated efforts to examining the effects of bioactive glass nanoparticles (BG-NPs) modified with boron, as previously mentioned. Their findings revealed that BG-NPs significantly promoted the proliferation of human dental pulp stem cells (hDPSCs), pinpointing an optimal bioactive glass concentration at 6.25 mg/mL. Moreover, the presence of boron in conjunction with NP-BG was associated with elevated levels of osteopontin, dentin sialophosphoprotein (DSPP), and type I collagen compared to samples containing NP-BG without boron, indicating enhanced osteogenic differentiation potential.

Conversely, Zhang et al. [12] embarked on a study to evaluate zinc oxide nanoparticles (NP-ZnO) in conjunction with various gutta-percha scaffolds, aiming to determine the cytotoxic effects of NP-ZnO on pulp cells. It was observed that dental pulp stem cells (DPSCs) cultured on gutta-percha scaffolds exhibited growth even in the absence of additional substances, leading to the conclusion that the integration of NP-ZnO into gutta-percha scaffolds encouraged the differentiation of dental pulp stem cells into various cell lineages, thus suggesting a potential avenue for enhancing regenerative outcomes in dental tissue engineering.

In the study conducted by Bellamy et al. [13], the focus was on elucidating the impact of chitosan nanoparticles (NP-CS) on stem cells from the apical papilla (SCAP). A critical observation from this research was the dual role of transforming growth factor-beta1 (TGF-b1) concentration in either stimulating or inhibiting SCAP activity. The investigation revealed a notable increase in the differentiation of apical papilla cells within the group treated with NP-chitosan in conjunction with TGF-b1 and a scaffold, compared to other experimental groups. Furthermore, the combination of NP-chitosan and the sustained release of TGF-b1 within a carboxymethylchitosan scaffold (CMCS) was identified as a potent stimulant for the enhancement of cell differentiation and migration processes in SCAP. This finding underscores the potential of NP-CS, especially when used in synergy with TGF-b1 and scaffolds, to significantly advance the regenerative capabilities of stem cells in dental tissue engineering.

In their 2018 investigation, Shen et al. [19] aimed to develop a framework composed of polylactic acid (PLA) and chitosan that would exhibit biocompatibility with periodontal tissue and bone. A particularly noteworthy discovery from this study was the dual effect of chitosan concentration on cell proliferation. Initially, the incorporation of chitosan was observed to enhance cell growth; however, at higher concentrations, it detrimentally affected the scaffold’s surface integrity, subsequently diminishing cell proliferation. This phenomenon aligns with findings from other studies [1,26], which have documented that when combined with other substances, chitosan nanoparticles (NPs) demonstrate improved efficacy. Specifically, in this context, the synergy between NP-chitosan and PLA outperformed the use of pure PLA nanofibers. Moreover, the composite of NP-chitosan and PLA notably promoted differentiation in bone marrow stem cells (BMSCs) and activated the TLR4 pathway in human periodontal ligament cells (hPDLCs), thereby highlighting its potential as an effective material for periodontal and bone tissue engineering.

Osmond et al. [20] investigated the use of nanoparticles of dicalcium phosphate dihydrate (NP-DCPD) and hydroxyapatite (NP-HA), as well as combinations of these nanoparticles with triethylene glycol dimethacrylate (TEGDMA). Their study revealed that DCPD nanoparticles exhibit osteoinductive potential. However, in cultures immersed with NP-HA, the initiation of human dental pulp stem cells’ (hDPSCs) differentiation occurred earlier than with NP-DCPD. In terms of bovine serum albumin (BSA) release, both NP-DCPD and NP-DCPD combined with TEGDMA demonstrated lower release rates. Conversely, among the HA variants, NP-HA exhibited a higher release rate, which was reduced when combined with TEGDMA.

In a related vein, Shrestha et al. [24] focused on the application of chitosan nanoparticles (NP-CS) loaded with dexamethasone (DEX) designed for either slow (I) or rapid (II) release mechanisms. These formulations were applied to the preparation of exposed root dentine surfaces, resulting in enhanced stem cells from the apical papilla (SCAP) adhesion. Notably, the group with rapid release dexamethasone (NP-CS + DEX II) exhibited higher expression levels of dentin sialophosphoprotein (DSPP) and dentin matrix protein 1 (DMP-1) compared to the NP-CS alone and the NP-CS + DEX I groups. Thus, the study concluded that a rapid release mechanism of DEX significantly influences SCAP cell activity, facilitating improved differentiation outcomes.

In a recent study conducted in 2022 by Saharkhiz et al. [14], the focus was placed on the biological effects of phytosomal curcumin (PC) nanoparticles on dental pulp stem cells (DPSCs). The research findings indicated that while high doses of PC-NPs (45–60 μM for 24 h) exhibited cytotoxic effects, lower concentrations (< 40 μM, across both 24 and 48 h) not only stimulated human DPSCs, but also adversely affected the expression of specific genes such as RelA, VCAM1, HLA-G5, and STAT3. Interestingly, the intervention with PC-NPs at these lower concentrations resulted in a notable enhancement in the expression of vascular endothelial growth factor A (VEGF-A) and dentin sialophosphoprotein (DSPP) over the same periods, thereby suggesting potential therapeutic benefits in dental tissue regeneration.

Liu et al. [17] embarked on a comparative study of the PAMAM dendrimer with non-collagenous dentin proteins (DNCP). Their investigation revealed no significant differences among the evaluated groups (P-PAMAM, DNCP, and Control) in terms of DPSC proliferation. However, both the P-PAMAM and DNCP groups exhibited an influence on mineralization, with DNCP showing a superior effect. The study concluded that while P-PAMAM dendrimers facilitate DPSC differentiation, they do not significantly impact cell proliferation at a concentration of 1 μg/mL, underscoring DNCP’s enhanced capacity for promoting dental tissue mineralization compared to P-PAMAM.

Hydroxyapatite nanoparticles (NP-HA) are increasingly recognized as one of the most promising materials for tissue regeneration. Alipour et al. [18] investigated the efficacy of a hydrogel composite comprising NP-HA, alginate, and gelatin (Alg + Gel + NP-HA) in modulating the activity of human dental pulp stem cells (hDPSCs). Their study revealed that hDPSCs encapsulated within this hydrogel demonstrated enhanced proliferative and differentiative capacities compared to other groups. This improvement was attributed partly to the presence of NP-HA, which also significantly boosted the expression of bone cell markers, including osteonectin, osteocalcin, and RUNX-2, surpassing the effects observed in the control groups.

In a parallel study, Huang et al. [21] explored the potential of mesoporous calcium silicate nanoparticles (MesoCS) in biomedical applications. The findings indicated that MesoCS nanoparticles possess the inherent capability to transport and release bioactive molecules, such as drugs, effectively. Additionally, these nanoparticles facilitated significant apatite precipitation, a feature highly beneficial for the mineralization of the extracellular matrix. This dual functionality not only hinders the proliferation of microorganisms, including bacteria, but also enhances the osteogenic differentiation of cells. The presence of osteogenic markers and the activity of alkaline phosphatase (ALP) were observed to increase, particularly when MesoCS was used in conjunction with fibroblast growth factor-2 (FGF-2), suggesting a synergistic effect that augments its regenerative potential.

In a detailed investigation by Liu et al. [23] on the potential of cationic polyethylene imine (PEI) and polyethylene glycol (PEG) nanoparticles (PEG-PEI) loaded with miR-146a and basic fibroblast growth factor (bFGF), it was discovered that while PEG-PEI nanoparticles possess the capability for drug release, their application in the context of lipopolysaccharide (LPS)-stimulated dental pulp stem cells (DPSCs) resulted in a reduction of cell growth and differentiation. Conversely, the combination of miR-146a with bFGF markedly enhanced cell proliferation and the stimulation of dental pulp cell differentiation. Notably, the administration of miR-146a and bFGF as separate entities did not yield significant effects. The study concluded that the synergistic interaction between bFGF and miR-146a significantly improved the capacity for cell differentiation and inflammation reduction, highlighting the potential of combining these agents for enhanced therapeutic outcomes.

Niu et al. [26] explored the effects of gold nanoparticles (AuNPs) on human periodontal ligament stem cells, particularly focusing on the activation of the p38 mitogen-activated protein kinase (MAPK) pathway and the resultant increase in RUNX-2 levels. Their findings confirmed that AuNPs effectively promote osteogenesis through the p38 MAPK pathway. The application of AuNPs was determined to significantly enhance cell differentiation, suggesting a promising avenue for the use of gold nanoparticles in the enhancement of osteogenic differentiation and the potential regeneration of periodontal tissues.

Lastly, studies conducted by Elshahat et al. [27] and Abdelaziz et al. [28] assessed the osteogenic differentiation and proliferation capabilities of SCAP through the application of nanoparticles. The research by Elshahat et al. utilized bioactive glass nanoparticles 45S5 (NBG), chitosan-coated nanohydroxyapatite (NHAP-CS), and hydroxyapatite nanoparticles (NHAP), while the study by Abdelaziz et al. evaluated bioactive glass nanoparticles 45S5 (NBG) and Neo MTA nanoparticles (NNMTA). The outcomes of both studies were strikingly similar, with the cells exhibiting CD44 and CD43 expressions but not CD45, indicating a non-hematopoietic origin. ALP expression was higher with NHAP-CS in the Elshahat et al. [27] study, and with NNMTA in the Abdelaziz et al. research, whereas RANKL expression scored higher with NHAP-CS and NBG, respectively, according to the studies. Regarding the proliferation of SCAP, the results were conclusive as both extracted a higher count of viable cells with NHAP-CS and NBG, respectively, in the studies, though the differences were not significant.

Despite the importance of assessing the toxicity of these materials, (such as the nanoparticles), only four of the selected studies investigate it. Saharkhiz et al. [14] observed that at high doses (45–60 μM for 24 h) the PC-NPs are cytotoxic, but at lower concentrations (<40 μM, for both 24 and 48 h) they stimulated the hDPSCs. Rao et al. [15] concluded that NP-BG + tideglusib, with improved mechanical and physical properties, did not cause toxicity to the pulp, favored the healing of lesions, and promoted the growth of hDPSCs. Zhang et al. [12] determined that NP-ZnO did not obtain conclusive results regarding toxicity. Lastly, Rad et al. [25], who focused their research on evaluating NPBG with boron determined that toxicity depended on the amount of boron added, thus being dose-dependent.

In light of the above, nanoparticles appear to have therapeutic potential with multiple possibilities and favorable effects for dental structures. The results obtained from this review highlight the need to continue researching nanoparticles to ascertain all their applications and adverse effects, especially in vivo studies in humans, and to verify if it will be possible, in the future, to adapt them to dental materials for regenerative purposes.

In addressing the constraints encountered during the execution of this systematic review, it is pertinent to underscore the limited volume of literature specifically dedicated to the topic of dental regeneration employing nanoparticles. This scarcity is primarily attributed to the predominant focus of existing research on the antimicrobial and antibiotic properties of nanoparticles, as revealed by the initial search outcomes. Despite conducting searches across multiple databases, a considerable proportion of the retrieved articles were duplicates, further compounding the challenge of sourcing relevant studies.

Moreover, the exclusion criteria applied in this review—specifically, the omission of reviews, articles not published in English, and in vivo studies involving both animal and human subjects—significantly narrowed the scope of included studies, thereby reducing the sample size. Additionally, the reliance on a solitary observer for the evaluation of studies introduces the possibility of variability in the interpretation of findings, which might have been mitigated through the involvement of multiple examiners. This methodological limitation suggests a potential area for enhancement in future systematic reviews through the incorporation of a broader examiner base to ensure a more comprehensive and nuanced analysis of the data.

## 5. Conclusions

The preponderance of research indicates a significant effect of NPs on DPSCs, suggesting their pivotal role in influencing pulp biology. Moreover, a consensus emerges from these studies regarding the NPs’ capability to modulate cell differentiation, with numerous investigations also highlighting their potential to enhance proliferation and mineralization. Such findings underscore the importance of NPs in initiating tissue regeneration processes. Furthermore, the therapeutic viability of NPs for dental regeneration is acknowledged, contingent upon the judicious selection of NP concentrations to circumvent cytotoxic effects, and the careful consideration of additional compounds, underscoring the nuanced application of NPs in regenerative dentistry.

## Figures and Tables

**Table 1:** Search method.

Databases	Search Field	Results
Medline (PubMed)	((“silver nanoparticles”) OR (nanoparticles)) AND ((endodontic) OR (“root canal treatment”) OR (“regenerative dentistry”))	744
Scopus	((“silver nanoparticles”) OR (nanoparticles)) AND ((endodontic) OR (“root canal treatment”) OR (“regenerative dentistry”))	407
Web of Science	((“silver nanoparticles”) OR (nanoparticles)) AND ((endodontic) OR (“root canal treatment”) OR (“regenerative dentistry”))	457

**Table 2:** Modified CONSORT checklist of items for reporting in vitro studies of dental materials [10].

Section/Topic	Checklist Item
**Abstract**	Item 1. Structured summary of trial design, methods, results, and conclusions
**Introduction** Background and objectives	Item 2a. Scientific background and explanation of rationale; Item 2b. Specific objectives and/or hypotheses
**Methods** Intervention	Item 3. Intervention for each group, including how and when it was administered, with sufficient detail to enable replication
Outcomes	Item 4. Completely defined, pre-specified primary and secondary measures of outcome, including how and when they were assessed
Sample size	Item 5. How sample size was determined
Randomization: sequence generation	Item 6. Method used to generate random allocation sequence
Allocation concealment mechanism	Item 7. Mechanism used to implement random allocation sequence (for example, sequentially numbered containers), describing any steps taken to conceal sequence until intervention was assigned
Implementation	Item 8. Who generated random allocation sequence, who enrolled teeth, and who assigned teeth to intervention
Blinding	Item 9. If done, who was blinded after assignment to intervention (for example, care providers, those assessing outcomes), and how
Statistical methods	Item 10. Statistical methods used to compare groups for primary and secondary outcomes
**Results** Outcomes and estimation	Item 11. For each primary and secondary outcome, results for each group, and estimated size of effect and its precision (for example 95% confidence interval)
**Discussion** Limitations	Item 12. Trial limitations, addressing sources of potential bias, imprecision, and, if relevant, multiplicity of analyses
**Other information** Funding	Item 13. Sources of funding and other support (for example suppliers of drugs), role of funders
Protocol	Item 14. Where full trial protocol can be accessed, if available

**Table 3:** Presentation of the different parameters of the studies selected for data extraction.

Author	Year	Type of Study	Control Group	Affected Tissue	Type of Nanoparticle	Effect
Moonesi Rad et al. [11]	2019	In vitro	hDPSC of third molars	Bone	Bioactive glass NPs + boron modified membrane	Promotes guided bone regeneration (GBR)
Zhang et al. [12]	2016	In vitro	DPSC of third molars	DPSC	ZnO NPs	Promotes DPSC differentiation
Bellamy et al. [13]	2016	In vitro	2.104 SCAP seeded	Apical papilla cells (SCAP)	Chitosan NP + TGF-B1	Promotes SCAP migration and differentiation
Saharkhiz et al. [14]	2022	In vitro	3rd human molars without caries	Dental pulp mesenchymal stem cells	NP of phytosomal curcumin	Promotes regeneration
Rao et al. [15]	2022	In vitro	Human dental pulp fibroblasts	Dentin-pulp complex	Bioactive glass + Tideglusib (tideglusib-BgNP)	Proliferation and migration of hDPSC (regeneration)
Moonesi Rad y cols et al. [16]	2019	In vitro	hDPSC of the third molar	Dentin	Bioactive glass NPs + modified boron	Promotes dentin regeneration
Liu et al. [17]	2022	In vitro	Human 3rd molars and premolars	Dental pulp stem cells	Phosphorylated P-PANAM	Odontogenic differentiation
Alipour et al. [18]	2021	In vitro	hDPSCs teeth	hDPSCs	Alg + Gel + NPHA	Odontogenic and osteogenic differentiation
Shen et al. [19]	2018	In vitro	hDPLSC	Periodontal tissue	Chitosan and PLA	Osteogenic proliferation and differentiation
Osmond et al. [20]	2021	In vitro	hDPSC	Pulp	NPDCPD/NPDCPD + TEGDMA NPHA/NPHA + TEGDMA	Direct pulp coating (DPL)
Huang et al. [21]	2017	In vitro	hDPSC of extracted human premolars	Pulp tissue	Mesoporous calcium silicate nanoparticles	Odontogenesis and biocompatible
Lim et al. [22]	2016	In vitro	hDPC Prof. Takata	Human dental pulp cells	Bioactive glass NPs + dexamethasone	Odontogenesis stimulation
Liu et al. [23]	2016	In vitro	Human premolars and 3ºM (DPC)	Dental pulp cells (DPC)	PEG–PEI NPs	Inflammatory response and regeneration
Shrestha et al. [24]	2016	In vitro	Extracted human teeth	SCAP	Dexamethasone + chitosan NP	Odontogenic differentiation
Rad et al. [25]	2018	In vitro	3rd human molars hDPSC	hDPSC	Boron-doped bioactive glass NPs	Odontogenic differentiation
Niu et al. [26]	2017	In vitro	Healthy extracted premolars hDPSC	hDPSC	Gold NPs (AFVBHuNPs)	Stimulates osteogenesis
Elshahat et al. [27]	2024	In vitro	3rd human molars	SCAP	Chitosan-coated nanohydroxyapatite and bioactive glass 45S5 NPs	Osteogenic differentiation and proliferation
Abdelaziz et al. [28]	2024	In vitro	3rd human molars	SCAP	Bioactive glass 45S5 NPs and Neo MTA	Osteogenic differentiation and proliferation

**Table 4:** Modified CONSORT Guide.

**Studies**	**[11]**	**[12]**	**[13]**	**[14]**	**[15]**	**[16]**	**[17]**	**[18]**	**[19]**
**Ítems**									
**1**	No	No	Yes	No	No	No	Yes	Yes	Yes
**2a**	No	Yes	Yes	Yes	Yes	No	Yes	Yes	Yes
**2b**	Yes	No	Yes	Yes	No	Yes	Yes	Yes	Yes
**3**	Yes	Yes	Yes	Yes	Yes	Yes	Yes	Yes	Yes
**4**	No	No	No	No	No	No	No	No	No
**5**	Yes	Yes	Yes	Yes	Yes	Yes	Yes	Yes	Yes
**6**	No	No	No	No	No	No	No	No	No
**7**	No	No	No	No	No	No	No	No	No
**8**	No	No	No	No	Yes*	No	No	No	No
**9**	No	No	No	No	No	No	No	No	No
**10**	Yes	No	Yes	Yes	Yes	Yes	Yes	Yes	Yes
**11**	Yes	Yes	Yes	Yes	Yes	Yes	Yes	Yes	Yes
**12**	No	No	No	No	Yes	No	Yes	No	Yes
**13**	Yes	Yes	Yes	Yes	Yes	Yes	Yes	Yes	Yes
**14**	No	No	No	Yes	No	No	No	Yes	Yes
**Bias%**	40%	33.33%	53.33%	53.33%	53.33%	40%	60%	60%	66.66%
**Studies**	**[20]**	**[21]**	**[22]**	**[23]**	**[24]**	**[25]**	**[26]**	**[27]**	**[28]**
**Ítems**									
**1**	No	Yes	No	Yes	Yes	No	No	Yes	Yes
**2a**	Yes	Yes	Yes	Yes	Yes	Yes	Yes	Yes	Yes
**2b**	No	Yes	Yes	Yes	Yes	No	Yes	Yes	Yes
**3**	Yes	Yes	Yes	Yes	Yes	Yes	Yes	Yes	Yes
**4**	No	No	No	No	No	No	No	No	No
**5**	Yes	Yes	No	Yes	Yes	Yes	Yes	Yes	Yes
**6**	No	No	No	No	No	No	No	No	No
**7**	No	No	No	No	No	No	No	No	No
**8**	No	No	No	No	No	No	No	No	No
**9**	No	No	No	No	No	No	No	No	No
**10**	Yes	Yes	Yes	Yes	Yes	Yes	Yes	Yes	Yes
**11**	Yes	Yes	Yes	Yes	Yes	Yes	Yes	Yes	Yes
**12**	No	No	No	Yes	No	No	Yes	No	No
**13**	Yes	Yes	Yes	Yes	Yes	Yes	Yes	Yes	Yes
**14**	No	No	No	No	No	No	No	No	No
**Bias%**	40%	53.33%	40%	60%	53.33%	40%	53.33%	53.33%	53.33%

## Data Availability

Not applicable.

## References

[1] Yazdanian M., Rahmani A., Tahmasebi E., Tebyanian H., Yazdanian A., Mosaddad S.A. (2021). Current and Advanced Nanomaterials in Dentistry as Regeneration Agents: An Update. Mini-Rev. Med. Chem..

[2] Zakrzewski W., Dobrzynski M., Zawadzka-Knefel A., Lubojanski A., Dobrzynski W., Janecki M., Kurek K., Szymonowicz M., Wiglusz R.J., Rybak Z. (2021). Nanomaterials Application in Endodontics. Materials.

[3] Sreenivasalu P.K.P., Dora C.P., Swami R., Jasthi V.C., Shiroorkar P.N., Nagaraja S., Asdaq S.M.B., Anwer M.K. (2022). Nanomaterials in Dentistry: Current Applications and Future Scope. Nanomaterials.

[4] Vasiliu S., Racovita S., Gugoasa I.A., Lungan M.A., Popa M., Desbrieres J. (2021). The Benefits of Smart Nanoparticles in Dental Applications. Int. J. Mol. Sci..

[5] Bapat R.A., Joshi C.P., Bapat P., Chaubal T.V., Pandurangappa R., Jnanendrappa N., Gorain B., Khurana S., Kesharwani P. (2019). The use of nanoparticles as biomaterials in dentistry. Drug Discov. Today.

[6] Khoroushi M., Khademi A.A., Dastgurdi M.E., Abdolrahimi M. (2016). Nanobiomaterials in endodontics. Nanobiomaterials in Dentistry: Applications of Nanobiomaterials.

[7] Bonilla-Represa V., Abalos-Labruzzi C., Herrera-Martinez M., Guerrero-Pérez M.O. (2020). Nanomaterials in Dentistry: State of the Art and Future Challenges. Nanomaterials.

[8] Siddiqui Z., Acevedo-Jake A.M., Griffith A., Kadincesme N., Dabek K., Hindi D., Kim K.K., Kobayashi Y., Shimizu E., Kumar V. (2022). Cells and material-based strategies for regenerative endodontics. Bioact. Mater..

[9] Page M.J., McKenzie J.E., Bossuyt P.M., Boutron I., Hoffmann T.C., Mulrow C.D., Shamseer L., Tetzlaff J.M., Akl E.A., Brennan S.E. (2021). The PRISMA 2020 statement: An updated guideline for reporting systematic reviews. BMJ.

[10] Faggion C.M. (2012). Guidelines for reporting pre-clinical in vitro studies on dental materials. J. Evid. Based Dent. Pract..

[11] Moonesi Rad R., Atila D., Evis Z., Keskin D., Tezcaner A. (2019). Development of a novel functionally graded membrane containing boron-modified bioactive glass nanoparticles for guided bone regeneration. J. Tissue Eng. Regen. Med..

[12] Zhang L., Yu Y., Joubert C., Bruder G., Liu Y., Chang C.C., Simon M., Walker S.G., Rafailovich M. (2016). Differentiation of Dental Pulp Stem Cells on Gutta-Percha Scaffolds. Polymers.

[13] Bellamy C., Shrestha S., Torneck C., Kishen A. (2016). Effects of a Bioactive Scaffold Containing a Sustained Transforming Growth Factor-beta 1 releasing Nanoparticle System on the Migration and Differentiation of Stem Cells from the Apical Papilla. J. Endod..

[14] Saharkhiz M., Ayadilord M., Emadian Razavi F., Naseri M. (2022). Effects of phytosomal curcumin treatment on modulation of immunomodulatory and pulp regeneration genes in dental pulp mesenchymal stem cells. Odontology.

[15] Rao A.C., Venkatesh K.V., Nandini V., Sihivahanan D., Alamoudi A., Bahammam H.A., Bahammam S.A., Zidane B., Bahammam M.A., Chohan H. (2022). Evaluating the Effect of Tideglusib-Loaded Bioactive Glass Nanoparticles as a Potential Dentine Regenerative Material. Materials.

[16] Moonesi Rad R., Pazarçeviren E., Ece Akgün E., Evis Z., Keskin D., Şahin S., Tezcaner A. (2019). In vitro performance of a nanobiocomposite scaffold containing boron-modified bioactive glass nanoparticles for dentin regeneration. J. Biomater. Appl..

[17] Liu J., Gao Y., Zhu X., Zhang Y., Xu H., Wang T., Zhang G. (2022). Phosphorylated PAMAM dendrimers: An analog of dentin non-collagenous proteins, enhancing the osteo/odontogenic differentiation of dental pulp stem cells. Clin. Oral Investig..

[18] Alipour M., Firouzi N., Aghazadeh Z., Samiei M., Montazersaheb S., Khoshfetrat A.B., Aghazadeh M. (2021). The osteogenic differentiation of human dental pulp stem cells in alginate-gelatin/Nano-hydroxyapatite microcapsules. BMC Biotechnol..

[19] Shen R., Xu W., Xue Y., Chen L., Ye H., Zhong E., Ye Z., Gao J., Yan Y. (2018). The use of chitosan/PLA nano-fibers by emulsion eletrospinning for periodontal tissue engineering. Artif. Cells Nanomed. Biotechnol..

[20] Osmond M.J., Krebs M.D. (2021). Tunable chitosan-calcium phosphate composites as cell-instructive dental pulp capping agents. J. Biomater. Sci. Polym. Ed..

[21] Huang C.-Y., Huang T.-H., Kao C.-T., Wu Y.-H., Chen W.-C., Shie M.-Y. (2017). Mesoporous Calcium Silicate Nanoparticles with Drug Delivery and Odontogenesis Properties. J. Endod..

[22] Lim H.C., Nam O.H., Kim M.J., El-Fiqi A., Yun H.M., Lee Y.M., Jin G.Z., Lee H.H., Kim H.W., Kim E.C. (2016). Delivery of dexamethasone from bioactive nanofiber matrices stimulates odontogenesis of human dental pulp cells through integrin/BMP/mTOR signaling pathways. Int. J. Nanomed..

[23] Liu L., Shu S., Cheung G.S., Wei X. (2016). Effect of miR-146a/bFGF/PEG-PEI Nanoparticles on Inflammation Response and Tissue Regeneration of Human Dental Pulp Cells. BioMed Res. Int..

[24] Shrestha S., Torneck C.D., Kishen A. (2016). Dentin Conditioning with Bioactive Molecule Releasing Nanoparticle System Enhances Adherence, Viability, and Differentiation of Stem Cells from Apical Papilla. J. Endod..

[25] Rad R.M., Alshemary A.Z., Evis Z., Keskin D., Altunbas K., Tezcaner A. (2018). Structural and biological assessment of boron doped bioactive glass nanoparticles for dental tissue applications. Ceram. Int..

[26] Niu C., Yuan K., Ma R., Gao L., Jiang W., Hu X., Lin W., Zhang X., Huang Z. (2017). Gold nanoparticles promote osteogenic differentiation of human periodontal ligament stem cells via the p38 MAPK signaling pathway. Mol. Med. Rep..

[27] Elshahat S., Elgendy A.A., Elsewify T. (2024). Osteogenic Differentiation and Proliferation of Apical Papilla Stem Cells Using Chitosan-Coated Nanohydroxyapatite and Bioactive Glass Nanoparticles. Eur. J. Dent. 5.

[28] Abdelaziz H., Mahran A.H., Elsewify T. (2024). Osteogenic differentiation and proliferation of apical papilla stem cells using nanoparticles of Neo MTA and bioactive glass. Saudi Dent. J..

